# Follistatin-like protein 1 sustains colon cancer cell growth and survival

**DOI:** 10.18632/oncotarget.25811

**Published:** 2018-07-27

**Authors:** Gerolamo Bevivino, Silvia Sedda, Eleonora Franzè, Carmine Stolfi, Antonio Di Grazia, Vincenzo Dinallo, Flavio Caprioli, Federica Facciotti, Alfredo Colantoni, Angela Ortenzi, Piero Rossi, Giovanni Monteleone

**Affiliations:** ^1^ Department of Systems Medicine, University of Tor Vergata, Rome, Italy; ^2^ Department of Pathophysiology and Transplantation, Università degli Studi di Milano, Milan, Italy; ^3^ Department of Experimental Oncology, European Institute of Oncology, Milan, Italy; ^4^ Department of Surgery, University of Tor Vergata, Rome, Italy

**Keywords:** FSTL1, colon tumorigenesis, cell death, cellular cycle, ERK1/2

## Abstract

Follistatin-like protein 1 (FSTL1) is a secreted glycoprotein, which controls several physiological and pathological events. FSTL1 expression is deregulated in many tumors, but its contribution to colon carcinogenesis is not fully understood. Here, we investigated the expression and functional role of FSTL1 in colorectal cancer (CRC). A significant increase of FSTL1 was seen in human CRC as compared to the surrounding non-tumor tissues and this occurred at both RNA and protein level. Knockdown of FSTL1 in CRC cells with a specific antisense oligonucleotide (AS) reduced expression of regulators of the late G1 phase, such as phosphorylated retinoblastoma protein, E2F-1, cyclin E and phospho-cyclin-dependent kinase-2, and promoted accumulation of cells in the G1 phase of the cell cycle thus resulting in diminished cell proliferation. Consistently, recombinant FSTL1 induced proliferation of normal intestinal epithelial cells through an ERK1/2-dependent mechanism. Cell cycle arrest driven by FSTL1 AS in CRC cells was accompanied by activation of caspases and subsequent induction of apoptosis. Moreover, FSTL1 knockdown made CRC cells more susceptible to oxaliplatin and irinotecan-induced death. Data indicate that FSTL1 is over-expressed in human CRC and suggest a role for this protein in favouring intestinal tumorigenesis.

## INTRODUCTION

Colorectal cancer (CRC), one of the most common cancers in both men and women, is the fourth leading cause of cancer-related mortality worldwide, mainly due to the lack of effective treatment of advanced disease [1]. Sporadic disease, in which there is no family history, accounts for approximately 70 percent of all CRCs. The pathogenesis of CRC is not fully understood but a large body of evidence suggests that CRC results from the accumulation of genetic and epigenetic modifications, which alter pathways regulating proliferation, apoptosis, and angiogenesis, and CRC cell growth and survival are favored by many molecules produced within the tumor microenvironment [2, 3].

Follistatin-like protein 1 (FSTL1), also named transforming growth factor (TGF)β1- stimulated clone 36 (TSC-36) or follistatin-related protein, is an extracellular secreted glycoprotein, which belongs to the BM-40/SPARC/osteonectin family. FSTL1 is widely expressed in human tissues and plays key functions in the regulation of cell survival, proliferation, differentiation and migration of many cell types [4]. FSTL1 is also a regulator of embryonic organogenesis [5] and exerts either detrimental or protective effects depending on the context and experimental setting analyzed [6, 7]. Disconnected (disco)-interacting protein 2 homolog A (DIP2A) is a member of the DIP2 protein family encoded by Dip2a gene, which mediates FSTL1 function and is expressed in neurons, mesenchymal, endothelial, smooth muscle cells and cardiomyocytes [8, 9]. Altered expression of FSTL1 has been described in malignancies [10–14], even though its contribution to carcinogenesis remains controversial as FSTL1 can either positively or negatively regulate cancer cell growth and survival [10–14]. For instance, FSTL1 overexpression has been associated with poor prognosis of glioblastoma [10], progression of prostate cancer [11] and bone metastasis [12]. Moreover, experimental studies have shown that FSTL1 inhibition promotes apoptotis of lung cancer cells [13]. In contrast, FSTL1 exerts tumor-suppressive actions in ovarian and endometrial carcinogenesis and its expression is down-regulated in metastatic clear-cell renal-cell carcinoma [14, 15]. Recent studies have also documented altered expression of FSTL1 in human CRC, but divergent results have been provided regarding the cell sources of the cytokine and its role in the regulation of CRC cell behavior [16–18]. Therefore, we here investigated whether CRC cells produce FSTL1 and assessed the contribution of FSTL1 in the growth and survival of CRC cells.

## RESULTS

### FSTL1 is upregulated in human sporadic CRC

**Figure 1:**
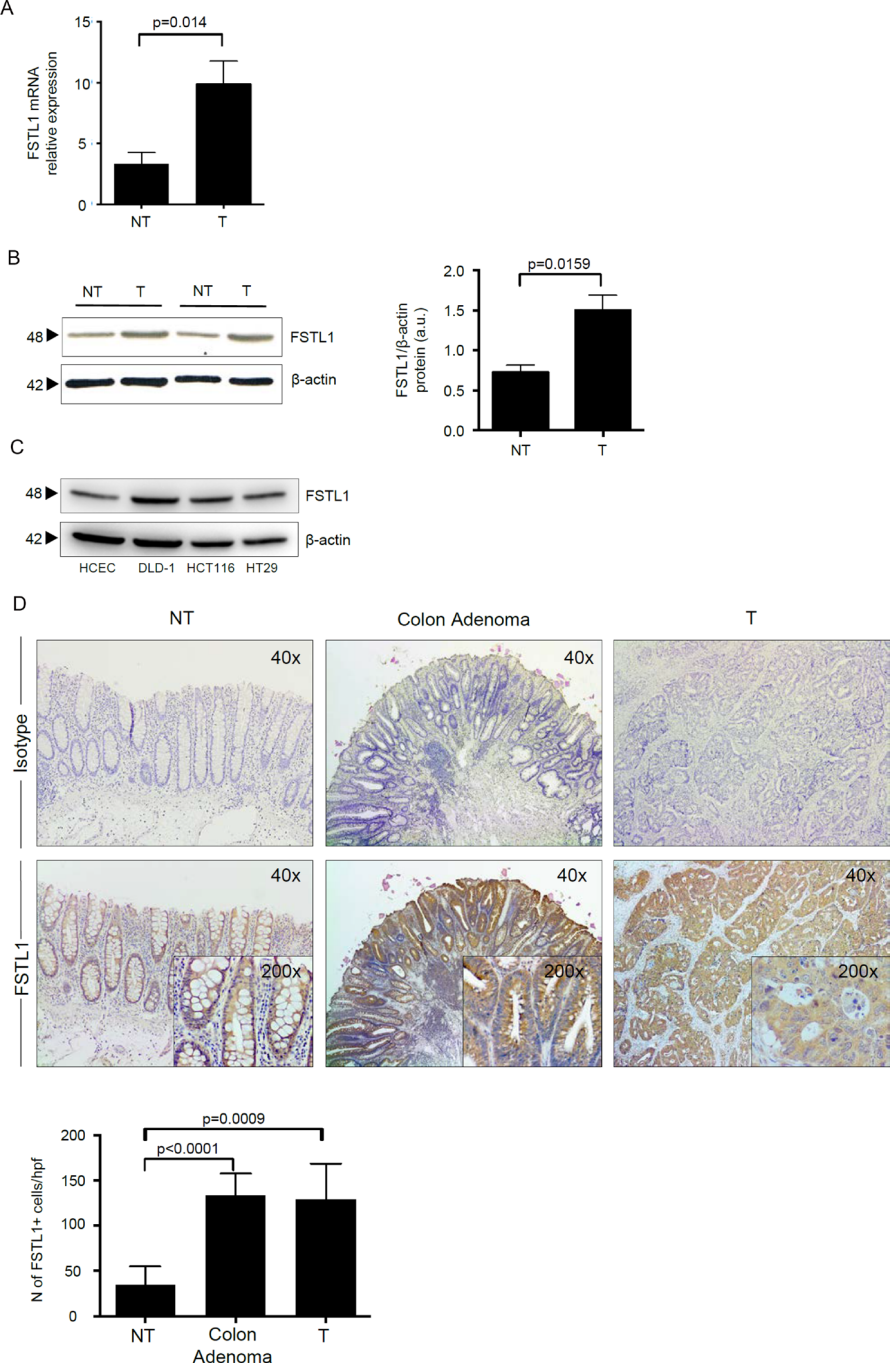
FSLT1 is overexpressed in human sporadic CRC and in CRC cell lines (**A**) FSTL1 RNA transcripts were evaluated in specimens taken from both tumoral (T) and non-tumoral (NT) areas of 12 patients with CRC by real-time PCR and levels were normalized to β-actin. Data are expressed as mean ± SEM of all samples. (**B**) Total proteins extracted from both tumoral (T) and non-tumoral (NT) areas of 12 patients with CRC were evaluated for FSTL1 expression by Western blotting. β-actin was used as loading control. The figure is representative of two separate experiments in which similar results were obtained. Right inset. Quantitative analysis of FSTL1/β-actin protein ratio in total extracts of T and NT tissues taken from 12 patients with CRC, as measured by densitometry scanning of Western blots. Values are expressed in arbitrary units (a.u.) and indicate the mean ± S.E.M. of all experiments. (**C**) Total proteins extracted from 3 CRC cell lines (i.e. DLD-1, HCT-116 and HT-29) and from the human colonic epithelial cell line (HCEC-1CT) were evaluated for FSTL1 expression by Western blotting. β-actin was used as loading control. One of 2 representative experiments in which similar results were obtained is shown. (**D**) FSTL1 immunostaining (x40 magnification) of colon adenoma sample taken from 1 patient who underwent endoscopic polipectomy and of surgical samples taken from tumoral (T) and non-tumoral (NT) areas of 1 patient with CRC. Staining with isotype control IgG is also shown. Higher magnification photomicrographs (×200) are shown in the insets. Panel shows the number of FSTL1-positive cells for high power field (hpf) of sections taken from 8 colon adenomas and non-tumoral (NT) and tumoral (T) areas of 8 patients with CRC. Data are expressed as mean ± SEM.

FSTL1 RNA transcripts were significantly increased in CRC samples as compared to non-tumoral samples (Figure 1A). Western blotting of total proteins extracted from freshly obtained samples and densitometry analysis of blots confirmed up-regulation of FSTL1 in the tumoral samples (Figure 1B). Consistent with the above data, FSTL1 was expressed in the CRC cell lines DLD-1, HCT-116 and HT-29 and to a lesser extent in HCEC-1CT, a normal epithelial colon cell line (Figure 1C). Moreover, immunostaining of normal and tumoral colonic sections showed a more pronounced expression of FSTL1 in adenoma and CRC cells as compared to normal colonic epithelial cells (Figure 1D). A diffuse and intense staining of FSTL1-positive cells was also seen in the lamina propria compartment of adenoma and CRC sections (Figure 1D).

### FSTL1 positively controls CRC cell growth

**Figure 2:**
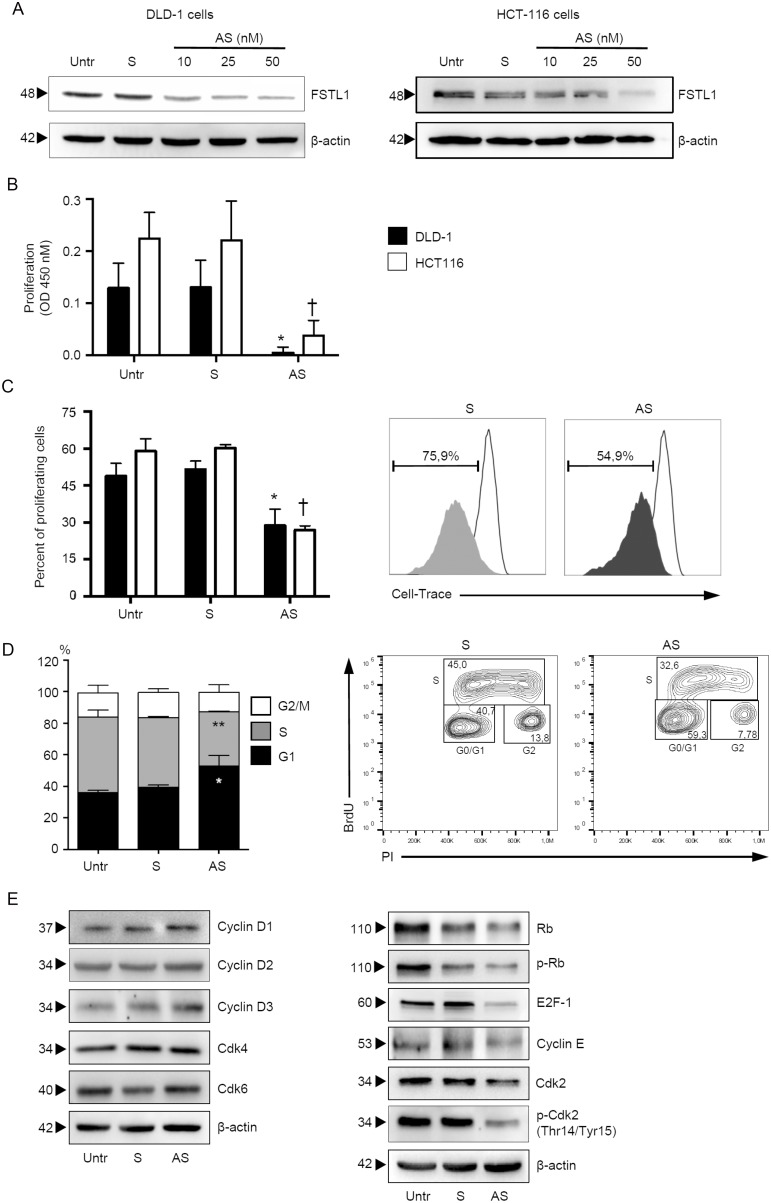
FSTL1 sustains CRC cells proliferation (**A**) FSTL1 antisense (AS) oligonucleotide downregulates FSTL1 protein expression in DLD-1 and HCT-116 cells. DLD-1 (left panel) and HCT-116 (right panel) cells were transfected with either FSTL1 sense (S) oligonucleotide (50 nM) or increasing doses (10–50 nM) of FSTL1 AS for 48 hours. FSTL1 and β-actin were analyzed by Western blotting. One of 3 representative experiments is shown. (**B**–**C**) FSTL1 AS inhibits the growth of CRC cell lines. DLD-1 and HCT-116 cells were either left untreated (Untr) or transfected with FSTL1 AS or FSTL1 S. (B) Cell proliferation was assessed by 5-bromodeoxyuridine (BrdU) assay kit. Data indicate mean ± S.E.M. of 3experiments (DLD-1: FSTL1 S-transfected cells versus FSTL1 AS-transfected cells, ^*^P < 0.001; HCT-116: FSTL1 S-transfected cells versus FSTL1 AS-transfected cells, ^†^*P* < 0.001). (C) Cell cultures were performed as above, and the percentage of proliferating cells was evaluated by flow cytometry. Data indicate mean ± S.E.M. of three experiments (DLD-1: FSTL1 S-transfected cells versus FSTL1 AS-transfected cells, ^*^P < 0.001; HCT-116: FSTL1 S-transfected cells versus FSTL1 AS-transfected cells, ^†^*P* < 0.001). Right panel. Representative plots are shown. (**D**) FSTL1 AS induces DLD-1 cells to arrest in G1 phase of cell cycle. DLD-1 cells were either left untreated (Untr) or transfected with FSTL1 S or AS. After 48 hours, cells were washed with PBS and cultured for further 24 hours. Cell cycle distribution was assessed by flow cytometry. Values are the percentages of cells in the different phases of cell cycle and indicate mean ± S.E.M. of 5 experiments. A significant increase in the number of cells that accumulate in G0/G1 phase (^*^P < 0.001) and a significant decrease in the number of cells in S phase (^**^P < 0.001) was seen in FSTL1 AS-transfected cells as compared with FSTL1 S-transfected cells. Right panel. Representative dot-plots showing the percentages of BrdU and/or PI-positive cells after 72 h. (**E**) FSTL1 knockdown in CRC cells reduces the levels of proteins involved in late G1 cell cycle phase. DLD-1 cells were either left untreated (Untr) or transfected with FSTL1 AS or FSTL1 S oligonucleotide. After 48 hours cells were washed with PBS and cultured for further 24 hours. Cyclin D1, cyclin D2, cyclin D3, Cdk4, Cdk6, Rb, p-Rb, E2F-1, cyclin E, Cdk2 and p-Cdk2 expression was assessed by Western blotting. β-actin was used as loading control. One of 3 representative experiments in which similar results were obtained is shown.

To determine whether FSTL1 regulates CRC cell proliferation, FSTL1 was inhibited in DLD-1 and HCT-116 cells with a specific antisense (AS) oligonucleotide (Figure 2A). Cells transfected with FSTL1 AS exhibited a significant reduction of 5-bromodeoxyuridine (BrdU) incorporation as compared to cells transfected with control oligonucleotide (Figure 2B). By flow-cytometry, we then showed that the percentage of proliferating cells was reduced by FSTL1 knockdown (Figure 2C). Analysis of cell cycle revealed that FSTL1 AS treatment significantly increased the number of cells in G1 phase and decreased the number of cells accumulated in S and G2 phases (Figure 2D and Supplementary Figure 1A), suggesting that DNA replication is inhibited following FSTL1 knockdown. In eukaryotic cells, the initiation of DNA replication and entry into mitosis are orchestrated by cyclin/cyclin-dependent kinase (CDK) complexes, which are formed at specific stages of the cell cycle [19]. Cdk4, Cdk6 and cyclin E-Cdk2 complexes, which sequentially phosphorylate the retinoblastoma protein (Rb), regulate transition from G1 phase to S phase [19]. Inhibition of FSTL1 resulted in a marked reduction of proteins involved in late G1 cell cycle phase, such as phosphorylated-Rb (p-Rb), E2F-1, cyclin E and phospho-Cdk2 (p-Cdk2) (Figure 2E, right panel and Supplementary Figure 1B, right panel), with no modification of early G1 phase proteins (i.e. cyclin D1, D2, D3, Cdk4, Cdk6) (Figure 2E, left panel and Supplementary Figure 1B, left panel).

### FSTL1 stimulates epithelial cell proliferation via ERK1/2-dependent pathway

**Figure 3:**
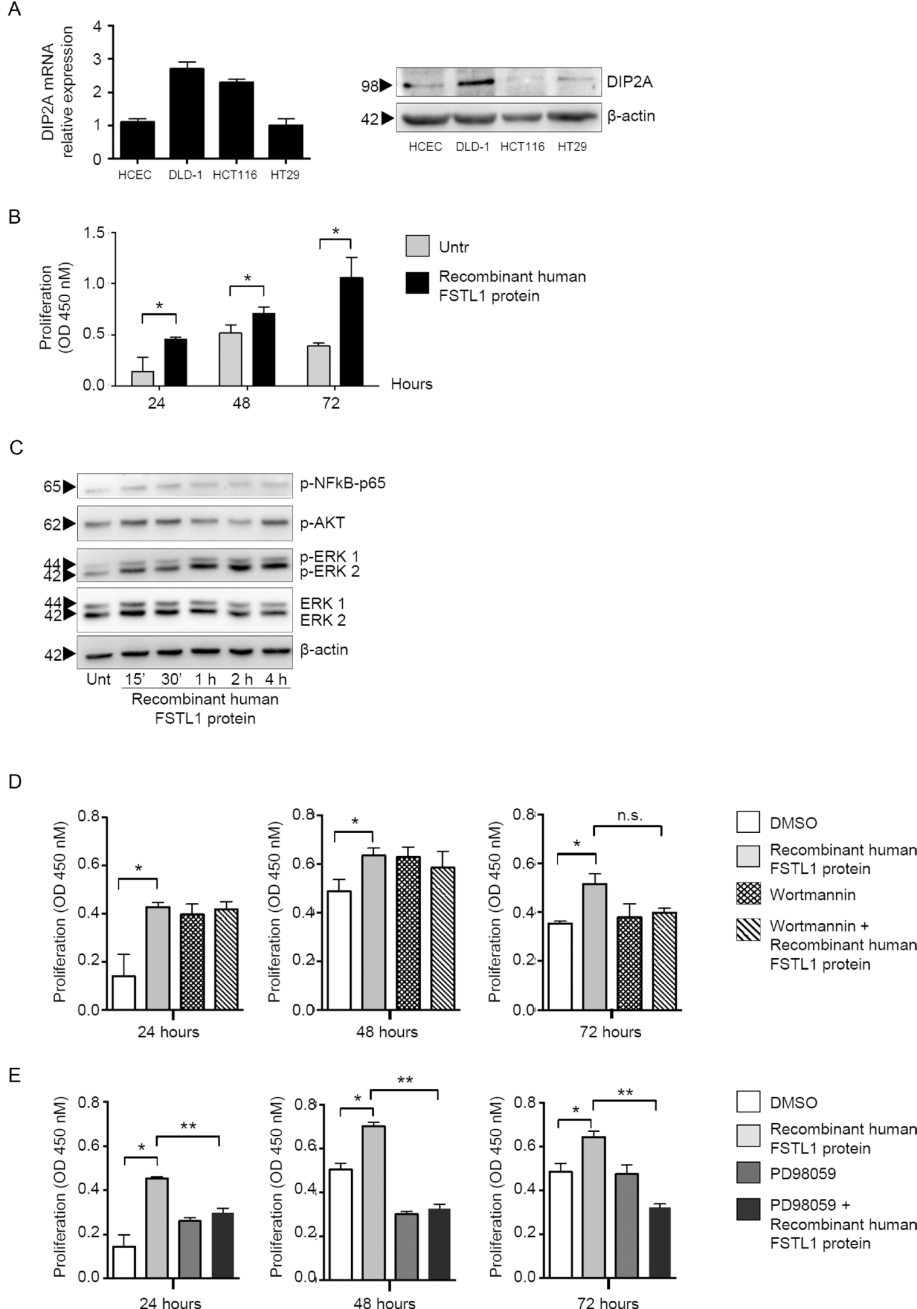
FSTL1 stimulates epithelial cell proliferation through an ERK-dependent mechanism (**A**) DIP2A is expressed in normal and neoplastic colon cell lines. Left panel. DIP2A RNA expression was evaluated in human colonic epithelial cell line (HCEC-1CT) and 3 CRC cell lines (i.e. DLD-1, HCT-116 and HT-29) by real-time PCR (left panel). Levels were normalized to β-actin. Data are expressed as mean ± SEM. Right panel. Total proteins extracted from the same cells were evaluated for DIP2A expression by Western blotting (right panel). β-actin was used as loading control. One of 2 representative experiments in which similar results were obtained is shown. (**B**) FSTL1 enhances cell proliferation. HCEC-1CT cells were stimulated with recombinant human FSTL1 protein for 24, 48 and 72 hours. Cell proliferation was assessed by using 5-bromodeoxyuridine (BrdU) assay kit. Data indicate mean ± S.E.M. of three experiments (^*^P < 0.05). (**C**) FSTL1 activates AKT and ERK1/2 pathways. HCEC-1CT cells were left either unstimulated or stimulated with recombinant FSTL1 at different time points. P-NF-kB/p65, p-AKT, p-ERK1/2 and ERK1/2 expression was assessed by Western blotting. β-actin was used as loading control. One of 3 representative experiments in which similar results were obtained is shown. (**D**–**E**) PD98059 significantly reduces FSTL1-driven cell growth. Cells were pre-treated with either wortmannin, an inhibitor of AKT (D), or PD98059, an inhibitor of ERK1/2 (E), prior to being stimulated with recombinant FSTL1 and cell proliferation was assessed by BrdU assay kit. Data indicate mean ± S.E.M. of 3 experiments (^*^P < 0.05; ^**^P < 0.0001).

In the subsequent studies, we dissected the basic mechanism by which FSTL1 positively regulates epithelial cell growth. By real-time PCR and Western blotting, we initially showed that DIP2A was expressed in normal and neoplastic colon cell lines (Figure 3A, left and right panel, respectively). Next, normal epithelial colon cells (i.e. HCEC-1CT) were stimulated with recombinant human FSTL1 protein for 24–72 hours and cell proliferation was evaluated as above. Treatment of cells with recombinant FSTL1 enhanced cell growth and this effect was evident at each time point (Figure 3B). To examine whether FSTL1 activates signalling pathways that control neoplastic cell proliferation, HCEC-1CT cells were left either unstimulated or stimulated with recombinant FSTL1 for different time points, and activation of NF-kB/p65, AKT and ERK1/2 MAP kinases was evaluated by Western blotting, using antibodies that recognise the active forms of these proteins. FSTL1 enhanced phosphorylation of AKT and ERK1/2 without affecting phosphorylation of NF-kB/p65 (Figure 3C). In parallel experiments, cells were pre-treated with either wortmannin, an inhibitor of AKT, or PD98059, an inhibitor of ERK1/2, prior to being stimulated with recombinant FSTL1. For these studies, we selected concentrations of wortmannin and PD98059, which selectively inhibit AKT and ERK1/2, respectively (not shown). Treatment of HCEC-1CT cells with Wortmannin did not affect FSTL1-induced cell proliferation (Figure 3D), while PD98059 significantly reduced FSTL1-driven cell growth (Figure 3E).

### FSTL1 knockdown induces CRC cell death through a caspase-dependent mechanism

**Figure 4:**
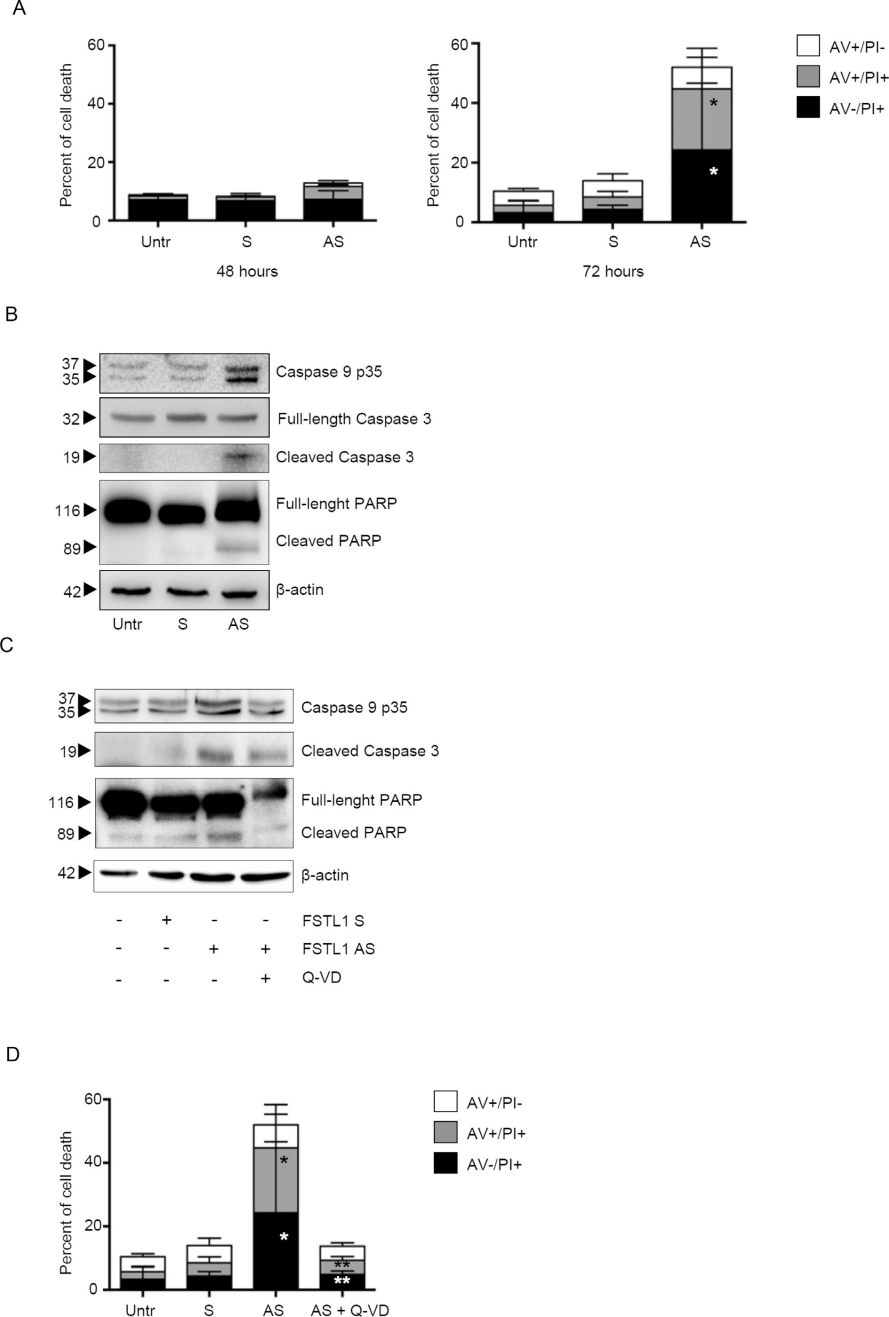
FSTL1 knockdown induces CRC cell death through a caspase-dependent mechanism (**A**) Inhibition of FSTL1 with AS induces DLD-1 cell death. DLD-1 were either left untreated (Untr) or transfected with FSTL1 S or AS (10 nM). After 48 h cells were washed with PBS and cultured for further 48 (left panel) or 72 (right panel) hours. Data indicate mean ± S.E.M. of three experiments (FSTL1 S-transfected cells versus FSTL1 AS-transfected cells, ^*^P < 0.001). (**B**) FSTL1 AS induces activation of caspases and poly ADP-ribose polymerase. Representative Western blots for cleaved caspase-9, full-length and cleaved caspase-3, full-length and cleaved PARP and β-actin in extracts of DLD-1 cells transfected with either FSTL1 S or AS. One of 3 representative experiments in which similar results were obtained is shown. (**C**) Pre-incubation of DLD-1 cells with pan-caspase inhibitor Q-VD-OPh (Q-VD) inhibits caspases and PARP activation. Representative Western blots for cleaved caspase-9, full-length and cleaved caspase-3, full-length and cleaved PARP in extracts of DLD-1 cells pre-incubated with Q-VD then transfected with either FSTL1 S or AS. One of 3 representative experiments in which similar results were obtained is shown. β-actin was used as loading control. (**D**) Pre-incubation of DLD-1 cells with Q-VD abolishes FSTL1 AS-induced cell death. Data indicate the percentage of cell death as assessed by flow cytometry analysis of Annexin V (AV) and/or propidium iodide (PI)-positive cells and are expressed as mean ± S.E.M of 4 experiments (FSTL1 S-transfected cells versus FSTL1 AS-transfected cells, ^*^P < 0.001; FSTL1 AS-transfected cells versus FSTL1 AS + Q-VD transfected cells, ^**^P < 0.001).

Since persistent block of cell cycle progression leads to cell death [20], we then evaluated whether FSTL1 inhibition affected the rate of cell survival. No significant change in the percentage of viable cells was seen after 48 hours of FSTL1 AS transfection (Figure 4A, left panel and Supplementary Figure 2A, left panel), thus indicating that FSTL1 AS-mediated CRC cell growth arrest was not secondary to cell death. Analysis of the fraction of Annexin V (AV)/propidium iodide (PI)-positive cells at later time points showed that accumulation of FSTL1-deficient CRC cells in G1 phase was accompanied by increased cell death at 72 hours (Figure 4A, right panel and Supplementary Figure 2A, right panel). To delineate the mechanism by which FSTL1 knockdown leads to cell death, we evaluated the expression of active caspases by Western blotting. Treatment of CRC cells with FSTL1 AS increased expression of active forms of caspase-9, caspase 3 and its direct substrate, poly ADP-ribose polymerase (PARP) (Figure 4B and Supplementary Figure 2B). Pre-incubation of DLD-1 and HCT-116 cells with a pan-caspase inhibitor, quinolyl-valyl- aspartate-OPh (Q-VD-OPh), prevented FSTL1 AS-driven caspase activation (Figure 4C and Supplementary Figure 2C) thus abolishing the FSTL1 AS-induced cell death (Figure 4D and Supplementary Figure 2D). These data indicate that FSTL1 knockdown induces CRC cell death through a caspase-dependent mechanism.

### FSTL1 knockdown augments the anti-cancer effects of oxaliplatin and irinotecan in CRC cells

**Figure 5:**
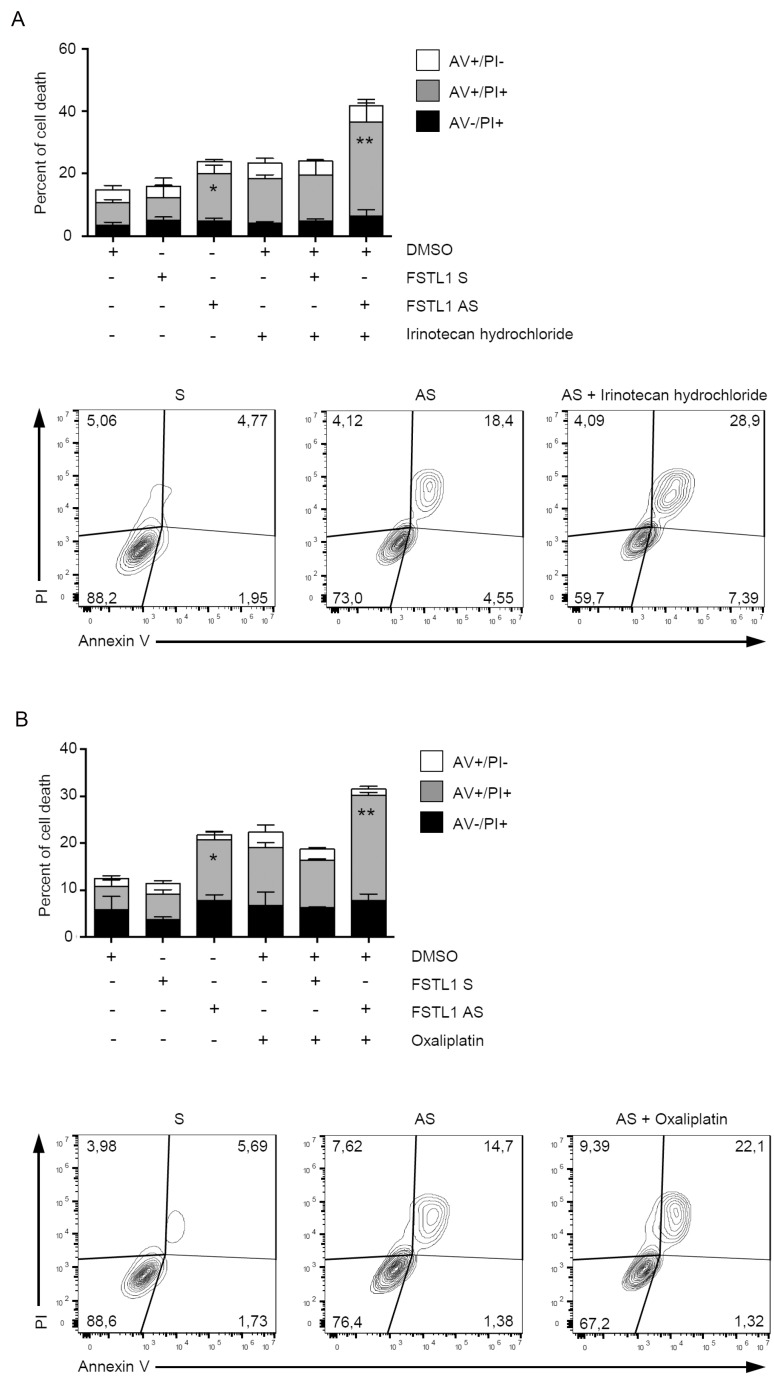
FSTL1 knockdown enhances the toxicity of chemotherapeutic drugs DLD-1 cells were transfected with either FSTL1 sense or AS (both used at 10 nM) for 48 hours and then stimulated with Irinotecan hydrochloride (**A**) and Oxaliplatin (**B**) (both used at a final concentration of 20 μM) or DMSO (vehicle) for further 72 hours. Data indicate the percentage of cell death as assessed by flow cytometry analysis of Annexin V (AV) and/or propidium iodide (PI)-positive cells and are expressed as mean ± S.E.M of 4 experiments (FSTL1 S-transfected cells versus FSTL1 AS-transfected cells, ^*^P < 0.05; FSTL1 AS-transfected cells versus FSTL1 AS + irinotecan hydrochloride or oxaliplatin transfected cells, ^**^P < 0.001). Representative dot-plots showing the percentages of AV- and/or PI-positive cells after 72 hours are also shown.

Chemotherapy-induced apoptosis is preferentially mediated by caspase activation [21]. To examine whether FSTL1 confers chemoresistance advantage to CRC cells, AV/PI flow cytometry analysis was performed on cisplatin- or -irinotecan-treated CRC cells cultured in the presence or absence of FSTL1 AS. Treatment of DLD-1 cells with FSTL1 AS increased the sensibility of CRC cells to oxaliplatin (Figure 5A) and irinotecan (Figure 5B).

## DISCUSSION

Accumulating evidence supports the notion that factors produced by immune cells and stromal cells sustain growth and survival of CRC cells [22, 23]. It is also known that CRC cells secrete additional factors that acting in a paracrine/autocrine manner amplify further proliferative and survival signals [24]. In this study, we show that FSTL1 is constitutively expressed in the normal colon and its expression is enhanced in CRC samples relative to surrounding non-tumor tissues. Immunoistochemical analysis of colonic sections showed that both cancer cells and lamina propria mononuclear cells express FSTL1. Up-regulation of the cytokine is also evident in sections of colon adenoma, raising the possibility that dysregulation of FSTL1 production occurs at early stages of colon carcinogenesis. Consistent with the above data, FSTL1 is detectable in various CRC cell lines. We show also that CRC cells express DIP2A, the known FSTL1 receptor, suggesting that FSTL1 might control CRC cell behavior. Indeed, knockdown of FSTL1 with a specific AS in CRC cell lines associates with arrest of cells in G1-phase of the cell cycle thus reducing cell growth. Time-course analysis shows also that block of cell cycle in FSTL1-deficient cells is followed by activation of caspases and induction of cell death. Moreover, FSTL1 knockdown enhances the susceptibility of CRC cells to chemotherapeutic drugs.

To delineate the basic mechanism by which FSTL1 controls CRC cell growth, we assessed the effect of recombinant FSTL1 on HCEC-1CT, as these cells express DIP2A. Treatment of HCEC-1CT with recombinant FSTL1 activates both AKT and ERK1/2 kinases and eventually promotes cell growth. However, studies with pharmacologic inhibitors of such kinases show that FSTL1-driven cell growth is mediated by ERK1/2 and not AKT. These data fit with recent studies showing a link between the inflammatory function of FSTL1 and activation of ERK1/2 [25].

Our findings confirm and expand on results of previous studies showing an abnormal expression of FSTL1 in CRC tissue. For example, by comparing the protein component of the whole-cell extracts and conditioned medium of primary cancer-associated fibroblasts (CAF) and normal fibroblasts isolated from mice with azoxymethane and dextrane sodium sulfate induced CRC, Torres and colleagues identified several proteins, including FSTL1, which were deregulated in tumoral samples [16]. Consistently, Chen *et al.* confirmed the increased expression of FSTL1 in CRC cell lines and primary fibroblasts, which were isolated from fresh surgical specimens of CRC samples [17]. By using co-culture experiments, the same authors showed that factors secreted by normal colonic fibroblasts and CAF inhibited CRC cell growth, an effect that could be abrogated by silencing of FSTL1 or transgelin (TAGLN) in fibroblasts. These findings would seem to suggest that stromal cell-derived FSTL1 and TAGLN might play important roles in the suppression of CRC cell growth. However, the survival analyses indicated that FSTL1 is not a significant prognosis factor for CRC and FSTL1 protein expression had no significant association with any clinic-pathological features in colon cancer patients, such as tumor size, grade, sex and phase [17]. Differences in the experimental procedures and systems used to evaluate functionally the role of FSTL1 in CRC cell growth could explain the discrepancies between data published by Chen *et al.* and our findings. The fact that knockdown of FSTL1 in various CRC cell lines abrogates neoplastic cell growth and stimulation of colonic cell lines with recombinant FSTL1 enhances cell proliferation suggests a direct and mitogenic effect of the cytokine on colonic epithelial cells. Consistent with our findings is the demonstration that co-expression of FSTL1 with p53 associates with poor prognosis, in patients with glioblastoma [10], thus reinforcing the concept that FSTL1 can sustain signals that amplify tumorigenesis. While this study was ongoing, Gu and colleagues showed that FSTL1 expression is significantly up-regulated in CRC tissues compared with the paired normal tissues and the higher FSTL1 expression in CRC associated with the infiltrating depth, lymph node metastasis and poor prognosis of the neoplasia [18]. Moreover, enhanced expression of FSTL1 distinctly increased cell migration and invasion *in vitro* and promoted liver metastasis of CRC *in vivo*.

In summary, the present study shows that FSTL1 is up-regulated in CRC tissue and activates in CRC cells signals that ultimately culminate in proliferation and survival. Therefore, our data provide evidence for FSTL1 being regarded as a promising target for therapeutic interventions in CRC patients.

## MATERIALS AND METHODS

### Patients and samples

Paired tissue samples were taken from the tumoral area and the macroscopically unaffected, adjacent, colonic mucosa of 20 patients who underwent colon resection for sporadic CRC at the Tor Vergata University Hospital (Rome, Italy) and used for FSTL1 evaluation by Western blotting and real-time PCR. Colon adenomas samples were taken from 6 patients who underwent to endoscopic polypectomy at the IRCCS Ospedale Maggiore Policlinico (Milan, Italy) and used for assessing FSTL1 by immunohistochemistry. Additional samples from the tumoral area and the macroscopically unaffected, adjacent, colonic mucosa of 8 CRC patients were used for assessing FSTL1 by immunohistochemistry. All patients received neither radiotherapy nor chemotherapy prior to undergoing surgery. The human studies were approved by the local Ethics committee and each patient gave written informed consent.

### Immunohistochemistry

All the reagents were from Sigma-Aldrich (Milan, Italy) unless specified. Immunohistochemistry was performed on formalin-fixed, paraffin-embedded sections of 6 colon adenomas and paired tumoral and non-tumoral samples of 8 CRC patients. The sections were deparaffinised and dehydrated through xylene and ethanol and the antigen retrieval was performed in Tris EDTA citrate buffer (pH 7.8) for 30 min in a thermostatic bath at 98° C (Dako Agilent Technologies, Glostrup, Denmark). Immunohistochemical staining was performed using a rabbit policlonal antibody directed against human FSTL1 (final dilution 1:500, Thermo Fisher Scientific, Waltham, MA) at room temperature for 1h followed by a biotin-free HRP-polymer detection technology with 3,3′diaminobenzidine (DAB) as a chromogen (MACH 4 Universal HRP-Polymer Kit, Biocare Medical, Pacheco, CA). The sections were counter-stained with haematoxylin, dehydrated and mounted. Isotype control IgG-stained sections were prepared under identical immunohistochemical conditions as described above, replacing the primary antibody with a purified mouse normal IgG control antibody (R&D Systems, Minneapolis, MN).

### Inhibition of FSTL1 by FSTL1 AS oligonucleotide

Custom LNA oligonucleotide targeting FSTL1 was provided by Exiqon (Vedbaek, Denmark). FSTL1 S or FSTL1 AS oligonucleotide was used at the final concentration of 10 nM or 50 nM for DLD-1 and HCT-116 cells respectively.

### Cell culture

The human CRC cell lines HCT-116, DLD-1 and HT-29, were obtained from the American Type Culture Collection (ATCC, Manassas, VA, USA) and the human colonic epithelial cell line, HCEC-1CT, was obtained from EVERCYTE GmbH (Vienna, Austria). Cells were maintained in McCoy's 5A (HCT-116, HT-29) and RPMI 1640 (DLD-1) medium, supplemented with 10% fetal bovine serum (FBS) and 1% penicillin/streptomycin (P/S) (both from Lonza, Verviers, Belgium) in a 37° C, 5% CO2, fully humidified incubator. HCEC-1CT were maintained in ColoUp medium (EVERCYTE GmbH) in a 37° C, 5% CO2, fully humidified incubator. Cell lines were recently authenticated by STR DNA fingerprinting using the PowerPlex 18D System kit according to the manufacturer's instructions (Promega, Milan, Italy). The STR profiles of all the cell lines matched the known DNA fingerprints. To investigate the role of FSTL1 in CRC cell growth and death, HCT-116 and DLD-1 cells were either left untreated or transfected with FSTL1 sense oligonucleotide or FSTL1 AS oligonucleotide for 48h using Opti-MEM medium and Lipofectamine 3000 reagent (both from Life Technologies, Milan, Italy) according to the manufacturer's instructions. Cells were then washed with Phosphate-buffered saline (PBS) and re-cultured with the respective fresh medium containing 0.05% Bovine Serum Albumin (BSA) for further 24–72 hours. In addition, DLD-1 cells were transfected with the specific FSTL1 AS or scrambled AS oligonucleotide (both used at the final concentration of 10 nM, Exiqon) for 48 hours and then stimulated with OXL and CPT-11 (all used at final concentration of 20 μM) or DMSO (vehicle) for further 72 hours. At the end, cell viability was evaluated by flow cytometry.

### Total protein extraction and Western blotting

Paired tissue samples of tumoral and non-tumoral areas and colon cell lines were lysed on ice in buffer containing 10 mM HEPES [pH 7.9], 10 mM KCl, 0.1 mM EthyleneDiamineTetraacetic Acid, 0.2 mM Ethylene Glycol-bis (β-aminoethyl ether)-N,N,N’,N’-Tetraacetic, and 0.5% Nonidet P40 supplemented with 1 mM dithiothreitol, 10 mg/ml aprotinin, 10 mg/ml leupeptin, 1 mM phenylmethylsulfonyl fluoride, 1 mM Na_3_VO_4_, and 1 mM NaF. Lysates were clarified by centrifugation at 4° C, 12.000 RCF for 30 minutes, and separated on sodium dodecyl sulphate (SDS)-polyacrylamide gel electrophoresis. Blots were incubated with the following antibodies: FSTL1 (1:1000 final diluition, Thermo Fisher Scientific), cyclin D1, cyclin D2, cyclin D3, Cdk2, p-Cdk2 (Thr14/Tyr15), Cdk4, Cdk6, Rb, E2F-1, cyclin E, caspase 9 (p35), full-length caspase 3, p-AKT, ERK1/2, p-ERK1/2 (1:500 final dilution, all from Santa Cruz Biotechnology INC, Santa Cruz, CA), p-Rb, cleaved caspase 3, PARP, p-NFkB (1:1000 final dilution, all from Cell Signaling Technology, Danvers, MA), DIP2A (1:500 final dilution, Novus Biologicals, Littleton, CO, USA), followed by horseradish peroxidase–conjugated secondary IgG monoclonal antibodies (all used at 1:20000 final dilution, Dako Agilent Technologies). After analysis, each blot was stripped and incubated with a mouse anti-human β-actin antibody (final dilution 1:5000) to ascertain equivalent loading of the lanes. Computer-assisted scanning densitometry (Chemidoc Touch Images, Biorad, Hercules, CA) was used to analyze the intensity of the immunoreactive bands.

### RNA extraction, cDNA preparation and real-time PCR

A constant amount of RNA (0,5 μg/sample) was retro-transcribed into complementary DNA (cDNA) and then 1 μl of cDNA/sample was amplified using the following conditions: denaturation 1 minute at 95° C; annealing 30 seconds, at 61° C for FSTL1, 61° C for DIP2A and 60° C for β-Actin, followed by 30 seconds of extension at 72° C. Primer sequences: FSTL1: forward, 5′-TCTGTGCCAATGTGTTTTGTGG-3′ and reverse, 5′-TGAGGTAGGTCTTGCCATTACTG-3′; DIP2A: forward, 5′-GGTGAACCTGTCATGTGTGC-3′ and reverse, 5′-CAGGTCCTTGAAGAGCTTGG-3′; β-actin: forward, 5′-AAGATGACCCAGATCATGTTTGAGACC-3′ and reverse, 5′-AGCCAGTCCAGACGCAGGAT-3′. Real-time-PCR was performed using the IQ SYBR Green Supermix (Bio-Rad) and RNA expression was calculated relative to the housekeeping β-actin gene on the base of the ΔΔCt algorithm.

### Analysis and quantification of DLD-1 and HCT-116 cells proliferation, cycle and death

Cell proliferation was assessed using CellTrace Violet Cell Proliferation Kit (Thermo Fisher Scientific), which covalently binds cell components to yield a fluorescence that is divided equally between daughter cells at each division. Briefly, cells were either left untreated or transfected with FSTL1 sense or FSTL1 antisense (AS) oligonucleotide (both used at 10 nM or 50 nM for DLD-1 and HCT-116 respectively). After 48 h, cells were washed with PBS and incubated with CellTrace according to the manufacturer's instructions. After 30 min, the medium was removed and fresh medium containing 0.05% BSA was added for further 24 h. At the end, cells were collected, washed twice with PBS, and then incubated with 5 mg/ml of PI for 15 min at 4 1C in the dark. CellTrace and/or PI-positive cells were determined by flow cytometry and the data were analyzed using Flow-Jo software (FlowJo LLC, Ashland, OR). Cell proliferation was also assessed using a commercially available BrdU assay kit (Roche Diagnostics, Mannheim, Germany). DLD-1 and HCT-116 cells were cultured in 96-well microplates and either left untreated or transfected with either FSTL1 sense oligonucleotide or FSTL1 AS oligonucleotide (both used at the concentrations specified above) for 48h. BrdU was added to the cell cultures 6 hours before the end of the treatment. BrdU-positive cells were evaluated by ELISA.

For analysis of cell cycle distribution, cells were either left untreated or transfected with either FSTL1 sense oligonucleotide or FSTL1 AS oligonucleotide (both used at 10 nM). After 48 h, cells were washed with PBS and recultured with fresh medium containing 0.05% BSA for further 24 h. At the end, cells were pulsed with 10 mol/L bromodeoxyuridine for 60 minutes, fixed in 70% cold ethanol, and stored at 20° C for at least 3 hours. Cells then were denatured in 2 mol/L HCl, and stained with anti–bromodeoxyuridine monoclonal antibody (Immunotech, Marseille, France) followed by fluorescein isothiocyanate–conjugated secondary anti-mouse immunoglobulin G (Molecular Probes, Milan, Italy). After staining with 100 g/mL PI, cells were analyzed by flow cytometry.

To score cell death, cells were either left untreated or transfected with either FSTL1 sense oligonucleotide or FSTL1 AS oligonucleotide (both used at 10 nM). After 48 h, cells were washed with PBS and re-cultured with fresh medium containing 0.05% BSA for further 48 or 72 h. In parallel, cells were pre-incubated in the presence or absence of Q-VD-OPh (R&D Systems, used at 10 μM) for 1 hour and then transfected with FSTL1 S or AS. Cells were then collected, washed twice in PBS, stained with FITC–annexin V (AV, 1:100 final dilution, Immunotools, Friesoyte, Germany) according to the manufacturer's instructions and incubated with 5 mg/ml PI for 30 min at 4 ° C, and their fluorescence was measured using FL-1 and FL-2 channels of FACSVerse (BD Biosciences) flow cytometer. Viable cells were considered as AV-/PI- cells, apoptotic cells as AV+/PI- cells, whereas secondary necrotic cells were characterized by AV+/PI+ staining.

### Analysis and quantification of HCEC-1CT cells proliferation

Cell proliferation was assessed by using a commercially available BrdU kit (Roche Diagnostics). Briefly, HCEC-1CT cells were cultured in 96-well microplates and stimulated with Wortmannin (final concentration 0,5 μM, Cell Signaling Technology) or PD98059 (final concentration 50 μM, Calbiochem, Burlington, Massachusetts) for 1 h, then treated with Human FSTL1 recombinant protein (final concentration 0,75 μg/ml, Sino Biological Inc., Beijing, China) and cultured for 24, 48 or 72 h. BrdU was added to the cell cultures 6 hours before the end of the treatment and the assay was evaluated by ELISA.

### Statistical analysis

Values are expressed as mean ± SEM and results analyzed using the two-tailed Student *t* test. GraphPad Prims6 (GraphPad Software, La Jolla, CA), was used for statistical and graphical data evaluations. Significance was defined as *P*-values < 0.05.

## SUPPLEMENTARY MATERIALS FIGURES AND TABLES


