# Supplementary material for: Follistatin-like protein 1 sustains colon cancer cell growth and survival
Source: Oncotarget. 2018 Jul 27;9(58):31278–90. doi: 10.18632/oncotarget.25811 (PMC6101290; doi:10.18632/oncotarget.25811)
Supplement: Supplementary file 1 [file oncotarget-09-31278-s001.pdf]

Follistatin-like protein 1 sustains colon cancer cell growth and survival

SUPPLEMENTARY MATERIALS

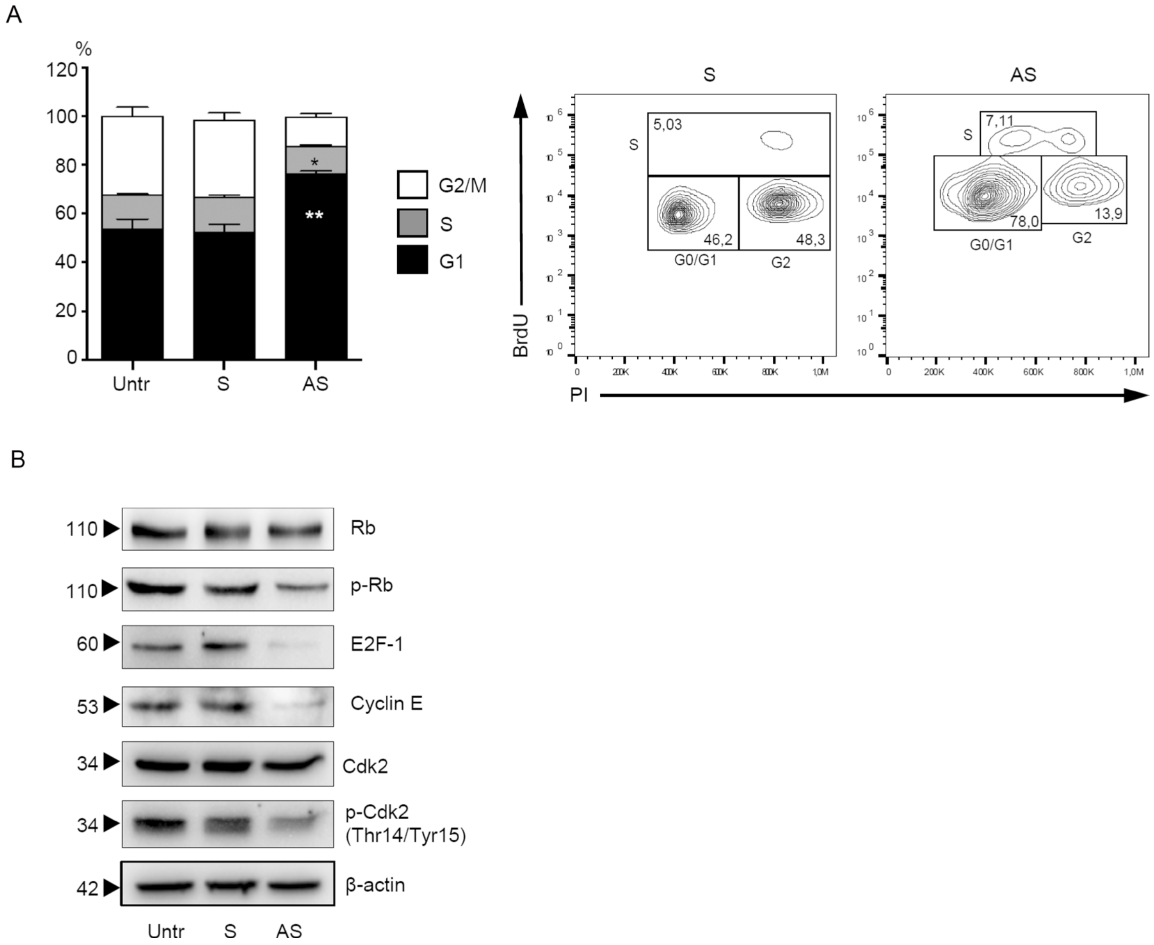

Supplementary Figure 1: FSTL1 antisense AS induces HCT-116 cells to arrest in G1 phase of cell cycle. (**A**) HCT-116 cells were either left untreated (Untr) or transfected with FSTL1 sense S or AS. After 24 hours, cells were washed with PBS and cultured for further 24 hours. Cell cycle distribution was assessed by flow cytometry. Values indicate the percentages of cells in the different phases of cell cycle and are expressed as mean ± S.E.M. of 5 experiments. A significant increase in the number of cells that accumulate in G0/G1 phase ($^{*}P < 0.001$) and a significant decrease in the number of cells in S phase ($^{**}P < 0.001$) was seen in FSTL1 AS-transfected cells as compared with FSTL1 S-transfected cells. Representative dot-plots showing the percentages of bromodeoxyuridine (BrdU) and/or PI-positive cells after 72 hours are shown. (**B**) FSTL1 knockdown in CRC cells reduces levels of proteins involved in late G1 cell cycle phase. HCT-116 cells were either left untreated (Untr) or transfected with FSTL1 AS or FSTL1 S oligonucleotide. After 24 hours, cells were washed with PBS and cultured for further 24 hours. Rb, p-Rb, E2F-1, cyclin E, Cdk2 and p-Cdk2 expression was assessed by Western blotting. β-actin was used as loading control. One of 3 representative experiments in which similar results were obtained is shown.

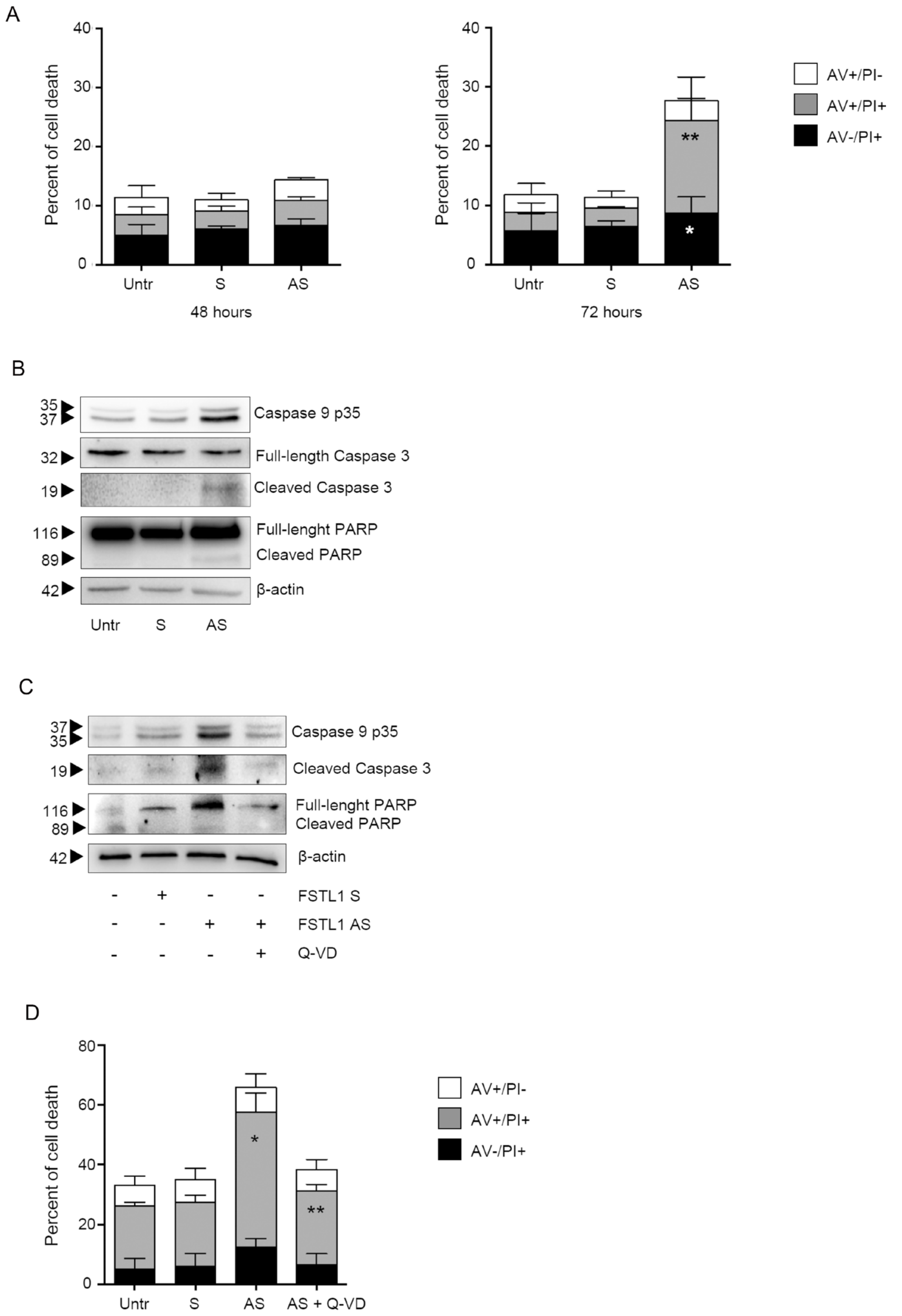

Supplementary Figure 2: FSTL1 knockdown induces CRC cell death through a caspase-dependent mechanism. (**A**) Inhibition of FSTL1 with antisense AS induces HCT-116 cell death. HCT-116 were either left untreated (Untr) or transfected with FSTL1 S or AS (10 nM). After 24 hours, cells were washed with PBS and cultured for further 48 (left panel) or 72 (right panel) hours. Data indicate mean ± S.E.M. of 3experiments (FSTL1 sense S-transfected cells versus FSTL1 AS-transfected cells, $^{*}P < 0.05$, $^{**}P < 0.001$). (**B**) FSTL1 AS induces activation of caspases and poly ADP-ribose polymerase. Representative Western blots for cleaved caspase-9, full-length and cleaved caspase-3, full-length and cleaved PARP and β-actin in extracts of HCT-116 cells transfected with either FSTL1 S or AS. One of 3 representative experiments in which similar results were obtained is shown. (**C**) Pre-incubation of HCT-116 cells with pan-caspase inhibitor Q-VD-OPh (Q-VD) inhibits caspases and PARP activation. Representative Western blots for cleaved caspase-9, full-length and cleaved caspase-3, full-length and cleaved PARP in extracts of HCT-116 cells pre-treated with Q-VD then transfected with either FSTL1 S or AS. One of 3 representative experiments in which similar results were obtained is shown. β-actin was used as loading control. (**D**) Pre-incubation of HCT-116 cells with Q-VD abolishes FSTL1 AS-induced cell death. Data indicate the percentage of cell death as assessed by flow cytometry analysis of Annexin V (AV) and/or propidium iodide (PI)-positive cells and are expressed as mean ± S.E.M of 4 experiments (FSTL1 S-transfected cells versus FSTL1 AS-transfected cells, $^{*}P < 0.001$; FSTL1 AS-transfected cells versus FSTL1 AS + Q-VD transfected cells, $^{**}P < 0.001$).
